# Anti-Apoptotic Effect of G-Protein-Coupled Receptor 40 Activation on Tumor Necrosis Factor-α-Induced Injury of Rat Proximal Tubular Cells

**DOI:** 10.3390/ijms20143386

**Published:** 2019-07-10

**Authors:** Chang Seong Kim, Soo Yeon Joo, In Jin Kim, Hoon-In Choi, Eun Hui Bae, Soo Wan Kim, Seong Kwon Ma

**Affiliations:** Department of Internal Medicine, Chonnam National University Medical School, Gwangju 61469, Korea

**Keywords:** G-protein-coupled receptor 40, apoptosis, tumor necrosis factor-alpha, angiotensin II type 1 receptor, ureteral obstruction, proximal kidney tubule

## Abstract

G-protein-coupled receptor 40 (GPR40) has an anti-apoptotic effect in pancreatic β-cells. However, its role in renal tubular cell apoptosis remains unclear. To explore the role of GPR40 in renal tubular apoptosis, a two-week unilateral ureteral obstruction (UUO) mouse model was used. The protein expression of GPR40 was decreased, while the Bax/Bcl-2 protein expression ratio, the expression of tumor necrosis factor (TNF)-α mRNA, and angiotensin II type 1 receptor (AT1R) protein were increased in mice with UUO. In vitro, pretreatment of rat proximal tubular (NRK52E) cells with GW9508, a GPR40 agonist, attenuated the decreased cell viability, increased the Bax/Bcl-2 protein expression ratio, increased protein expression of cleaved caspase-3 and activated the nuclear translocation of nuclear factor-κB (NF-κB) p65 subunit induced by TNF-α treatment. TNF-α treatment significantly increased the expression of AT1R protein and the generation of reactive oxygen species (ROS), whereas GW9508 treatment markedly reversed these effects. Pretreatment with GW1100, a GPR40 antagonist, or silencing of GPR40 in NRK52E cells promoted the increased expression of the cleaved caspase-3 protein by TNF-α treatment. Our results demonstrate that decreased expression of GPR40 is associated with apoptosis via TNF-α and AT1R in the ureteral obstructed kidney. The activation of GPR40 attenuates TNF-α-induced apoptosis by inhibiting AT1R expression and ROS generation through regulation of the NF-κB signaling pathway.

## 1. Introduction

G-protein-coupled receptor 40 (GPR40), also known as free fatty acid receptor 1, is a cell surface receptor highly expressed in pancreatic β-cells, intestine and enteroendocrine cells of the gastrointestinal tract, taste cells, immune cells, splenocytes, and brain cells [1,2,3,4]. We previously demonstrated that GPR40 is also expressed in a renal tubular epithelial cell line, and in rat and mouse kidneys [5,6].

GPR40 couples with a G protein α-subunit of the Gq family [7], and its activation in pancreatic islets induces the activity of phospholipase C and the hydrolysis of inositol lipids, as well as the intracellular calcium levels [8,9]. Computational and experimental studies have suggested that H137, R183, N244, and R258 are sites for agonist recognition and are directly involved in interactions with the ligand [10]. GPR40 agonists play a protective role against apoptosis of pancreatic β-cells, which could provide a treatment for diabetes by increasing insulin release [11,12]. Although GPR40 is involved in diverse physiology processes, the underlying apoptotic signaling pathways associated with GPR40 have not been clearly elucidated. A previous study showed that GPR40 overexpression ameliorates intestinal inflammation by the down-regulation of tumor necrosis factor (TNF) receptor 2 and the suppression of the NF-κB signaling pathway [13]. The stimulation of GPR40 also attenuates the induction of inflammatory cytokines and chemokines in TNF-α and interferon-γ-treated keratinocytes [14]. Pretreatment with GW9508, a GPR40 agonist, ameliorated the cisplatin-induced apoptotic death of human renal proximal tubular epithelial cells by inhibiting the generation of reactive oxygen species (ROS), pro-apoptotic proteins, and the activation of the Src/epidermal growth factor receptor/extracellular signal-regulated kinase signaling pathway, and the nuclear factor-κB (NF-κB) [5].

Tubular atrophy is the common characteristic feature of chronic kidney disease (CKD) and is superior to glomerular pathology as a predictor of CKD progression [15]. Tubular epithelial cell apoptosis has been implicated in tubular atrophy in studies using mouse models of focal and segmental glomerulosclerosis and a sublethal dose of diphtheria toxin [16,17]. Moreover, increased levels of TNF-α and angiotensin II type 1 receptor (AT1R) may play critical roles in the pathogenesis of renal tubular cell apoptosis [18,19]. Thus, GPR40 may contribute to anti-apoptotic effects by suppression of the TNF-α signaling pathway.

In this study, we determined whether the expression of GPR40 was changed in a ureteral obstructed kidney model and was associated with apoptosis. Furthermore, we investigated the effects of GPR40 activation on the pathogenesis of apoptosis induced by TNF-α treatment in rat proximal tubular (NRK52E) kidney cells.

## 2. Results

### 2.1. GPR40 Expression in the Ureteral Obstructed Kidney

**Figure 1:**
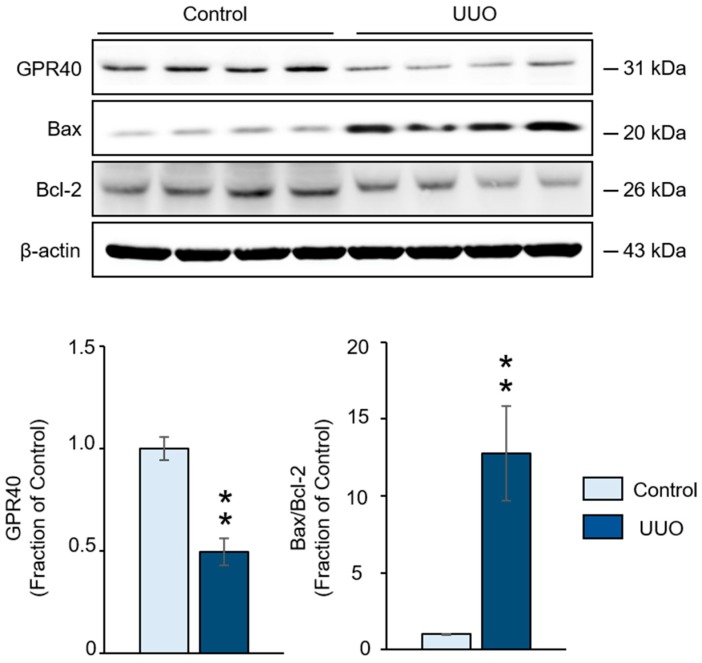
Western blot analyses of G-protein-coupled receptor 40 (GPR40), Bax and Bcl-2 proteins in the ureteral obstructed kidney of mice with unilateral ureteral obstruction (UUO). Representative Western blot and quantitative data are presented. The data are representative of independent experiments. The data are expressed as means ± SEM. *n* = 4. ** *p* < 0.01 compared with the control.

Following unilateral ureteral obstruction (UUO) for 2 weeks, expression of the GPR40 protein was significantly decreased, while the Bax/Bcl-2 protein ratio was increased in the obstructed kidney compared with that in the control (Figure 1).

**Figure 2:**
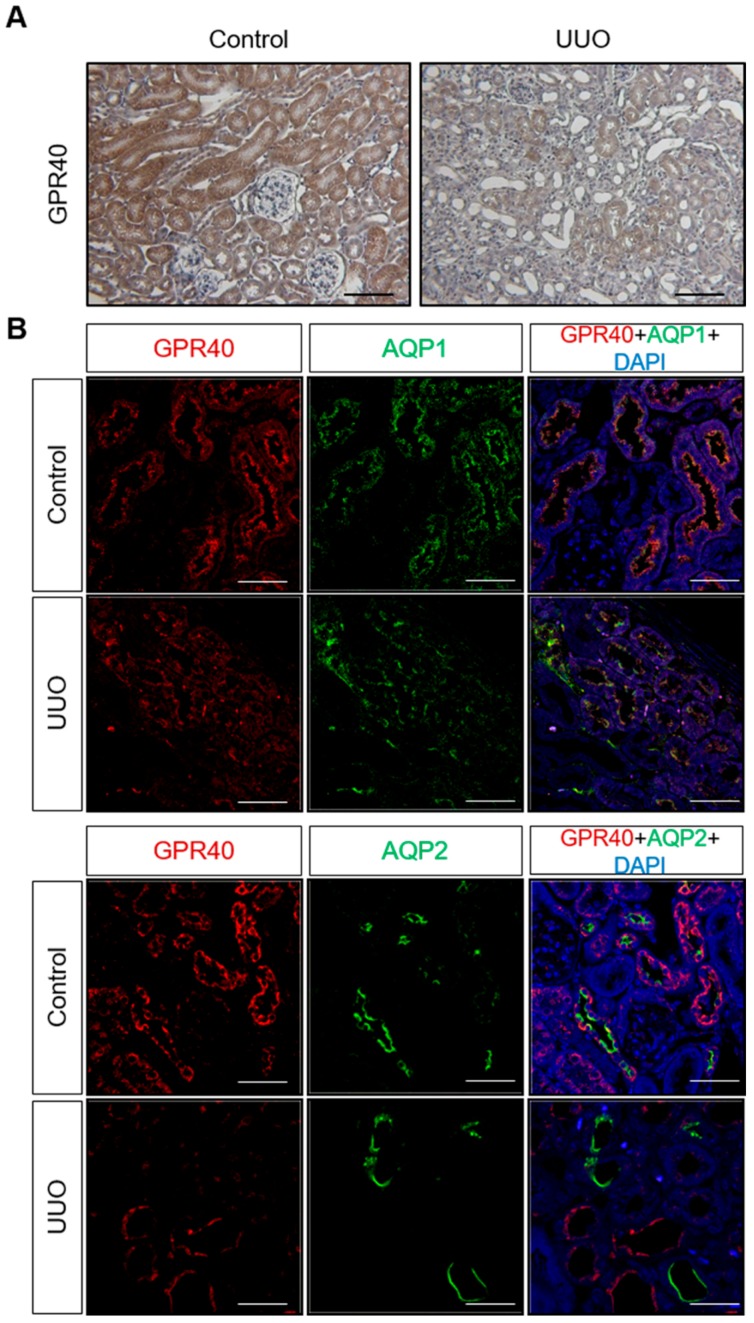
(**A**) Immunohistochemistry of G-protein-coupled receptor 40 (GPR40) expression in the control and ureteral obstructed kidneys of unilateral ureteral obstruction (UUO) mice (original magnification ×200). Bar = 100 μm. (**B**) Immunofluorescence staining of GPR40 in aquaporin-1 (AQP1)- and AQP2-positive renal tubules of the control and ureteral obstructed kidneys (original magnification ×400). Bar = 50 μm.

Immunoreactive GPR40 was expressed in renal tubules of the control kidney. However, decreased immunostaining of GPR40 with tubular atrophy was observed in the ureteral obstructed kidney (Figure 2A).

**Figure 3:**
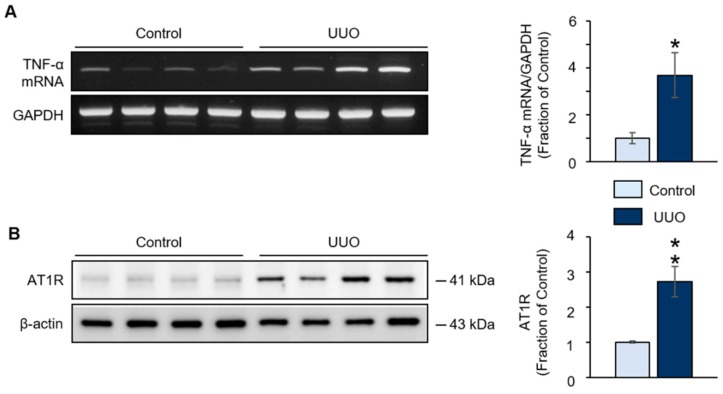
(**A**) Reverse transcription polymerase chain reaction (RT-PCR) analysis of tumor necrosis factor (TNF)-α mRNA and (**B**) Western blot analysis of angiotensin II type 1 receptor (AT1R) protein levels in the ureteral obstructed kidney of mice with unilateral ureteral obstruction (UUO). Representative RT-PCR and Western blot, and quantitative data are presented. The data are representative of independent experiments. The data are expressed as means ± SEM. *n* = 4. * *p* < 0.05 compared with the control. ** *p* < 0.01 compared with the control.

Immunofluorescence staining revealed decreased immunoreactivity of GPR40 in aquaporin-1 (AQP1)- and AQP2-positive renal tubules of the ureteral obstructed kidney (Figure 2B). In addition, the expression of TNF-α mRNA and the AT1R protein was increased in the ureteral obstructed kidney compared to control mice (Figure 3A,B).

### 2.2. Effects of a GPR40 Agonist on Apoptotic Signaling Induced by TNF-α

**Figure 4:**
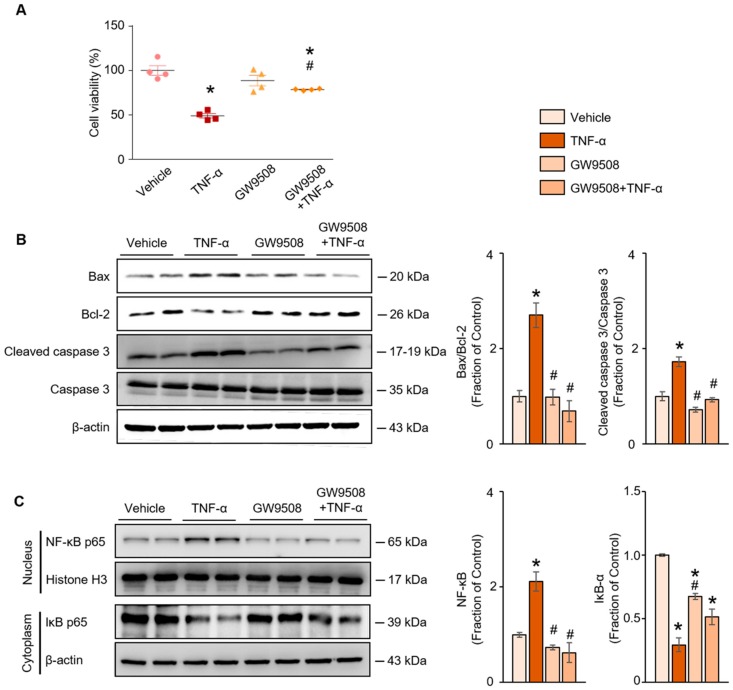
(**A**) Cell viability assay, (**B**) Western blot analyses of the levels of pro-apoptotic proteins (Bax, Bcl-2 and cleaved caspase-3) and (**C**) nuclear translocation of the NF-κB p65 subunit in tumor necrosis factor (TNF)-α-treated rat proximal tubular (NRK52E) cells with or without pretreatment with GW9508, a G-protein-coupled receptor 40 agonist. The representative Western blot and the quantitative data are presented. Shown are representative blots from three separate experiments with similar results. The data are expressed as means ± SEM. * *p* < 0.05 compared with vehicle-treated NRK52E cells. ^#^ *p* < 0.05 compared with TNF-α-treated NRK52E cells.

Next, we performed in vitro studies to explore the effect of GPR40 on apoptosis and the downstream signaling pathways activated in response to TNF-α. The WST-1 assay revealed that treatment of NRK52E cells with TNF-α (20 ng/mL) decreased their viability compared with vehicle-treated cells. Pretreatment with GW9508, a small-molecule GPR40 agonist, attenuated the decreased cell viability caused by TNF-α (Figure 4A). Treatment with TNF-α increased the Bax/Bcl-2 protein ratio and the cleaved caspase-3 level, compared with vehicle-treated cells. These changes were ameliorated by pretreatment with GW9508 (Figure 4B). As shown in Figure 4C, treatment with TNF-α also caused nuclear translocation of the NF-κB p65 subunit, which was counteracted by pretreatment with GW9508.

**Figure 5:**
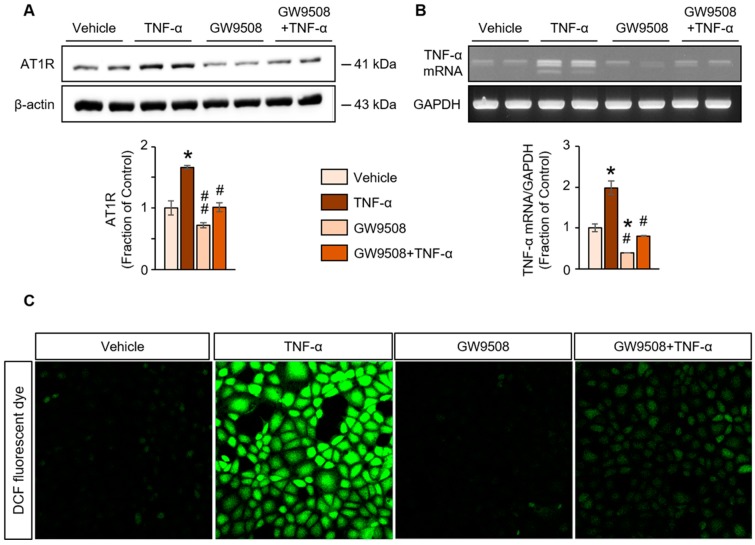
(**A**) Western blot analysis of angiotensin II type 1 receptor (AT1R) protein, (**B**) reverse transcription polymerase chain reaction (RT-PCR) analysis of tumor necrosis factor (TNF)-α mRNA and (**C**) generation of reactive oxygen species in TNF-α-treated rat proximal tubular (NRK52E) cells with or without pretreatment with GW9508, a G-protein-coupled receptor 40 agonist. The representative Western blot and RT-PCR, and the quantitative data are presented. Shown are the representative blots from three separate experiments with similar results. The data are expressed as means ± SEM. * *p* < 0.05 compared with vehicle-treated NRK52E cells. ^#^ *p* < 0.05 compared with TNF-α-treated NRK52E cells. ^##^ *p* < 0.01 compared with TNF-α-treated NRK52E cells.

Alterations in AT1R protein expression, TNF-α mRNA levels and ROS generation were examined to determine the mechanisms of TNF-α-induced tubular injury. All of these factors were increased by treating NRK52E cells with TNF-α. Notably, we observed that these TNF-α-stimulated changes were attenuated by pretreatment with GW9508 (Figure 5A–C).

**Figure 6:**
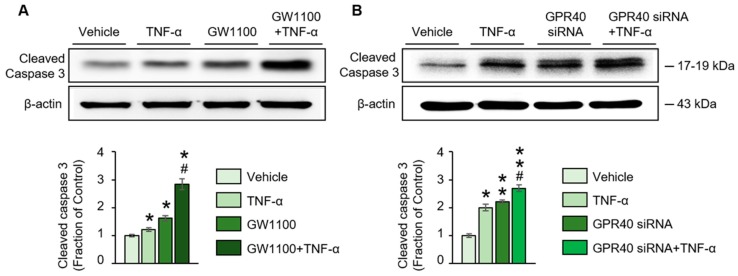
(**A**) Western blot analysis of the expression of the cleaved caspase-3 protein in tumor necrosis factor (TNF)-α-treated rat proximal tubular (NRK52E) cells with or without pretreatment with GW1100, a G-protein-coupled receptor 40 (GPR40) antagonist, or (**B**) GPR40 siRNA transfection. The representative Western blot and the quantitative data are presented. Shown are the representative blots from three separate experiments with similar results. The data are expressed as means ± SEM. * *p* < 0.05 compared with the vehicle-treated NRK52E cells. ** *p* < 0.01 compared with the vehicle-treated NRK52E cells. ^#^ *p* < 0.05 compared with the TNF-α-treated NRK52E cells.

Lastly, pretreatment with GW1100, a GPR40 antagonist, or silencing GPR40 in NRK52E cells with siRNA, markedly augmented the increased levels of cleaved caspase-3 caused by TNF-α treatment (Figure 6A,B). These data suggest that inhibiting GPR40 promotes renal tubular cell apoptosis, induced through the TNF-α signaling pathway. Thus, the activation of GPR40 plays an essential anti-apoptotic role.

## 3. Discussion

Urinary tract obstruction has been recognized as an experimental model of CKD because the same cellular and molecular events that characterize the progression of CKD also occur in the ureteral obstructed kidney of rats and mice with UUO [19,20]. Tubular cell apoptosis is pathogenetically related to the tubular atrophy and renal tissue loss that occurs in prolonged ureteral obstruction [21]. Therefore, we investigated whether GPR40, which is highly expressed in the kidney, has an association with apoptosis in the obstructed mouse kidney after UUO. Our results demonstrate that the expression of GPR40 was decreased in the obstructed kidney of the UUO mouse model. Furthermore, the activation of GPR40 by GW9508 attenuated renal tubular apoptosis by inhibiting ROS generation and the NK-κB signaling pathway, by regulating TNF-α and AT1R expression. Thus, GPR40 appears to regulate renal apoptosis through the inhibition of TNF-α/AT1R and NF-κB signaling pathways.

GPR40 was identified as an orphan 7-transmembrane G-protein-coupled receptor [22]. It functions as a cell surface receptor for medium- and long-chain fatty acids, and is primarily expressed in the brain and the pancreas [1,2]. Interestingly, we previously demonstrated that GPR40 is also expressed in the kidney [6]. Furthermore, the GPR40 protein level is decreased in the kidneys of rats treated with cisplatin in association with an increase in the serum creatinine level and the renal Bax/Bcl-2 protein ratio [5].

The GPR40 protein and mRNA are expressed in the renal tubular epithelial cell line, LLCPKcl4, and mouse kidney, as well as the pancreas and the brain [6]. Immunoblotting revealed that the GPR40 protein was expressed more abundantly in the cortex and the outer stripe of the outer medulla than in the inner stripe of the outer medulla and the inner medulla of the mouse kidney. In situ hybridization showed that GPR40 mRNA was localized to the renal cortical tubules of mouse kidneys, including the cortical collecting duct [6]. Nevertheless, the renal expression and function of GPR40 were not established in models of chronic kidney injury.

In the present study, we demonstrated that the expression of the GPR40 protein was decreased in the ureteral obstructed mouse kidney compared with the control kidney, and was related to the increased Bax/Bcl-2 protein ratio and tubular atrophy. The GPR40 protein was expressed in both AQP1- and AQP2-positive renal tubules. These results suggest that a decreased renal tubular expression of GPR40 may be related to the pathogenesis of apoptotic tubular injury in the ureteral obstructed kidney.

Ureteral obstruction induces the production of renal TNF-α and AT1R, renal tubular cell apoptosis, caspase activity, and NF-κB activity, and increases Bax and decreases Bcl-2 expression, all of which are ameliorated by neutralizing TNF-α [23,24,25]. The activation of pro-apoptotic proteins by a large number of factors, such as angiotensin II, TNF-α, ROS, and NF-κB, plays an important role in the pathogenesis of the apoptotic cell death of renal tubules in the ureteral obstructed kidney. Furthermore, these factors that initiate apoptosis interact with each other [21]. In addition, angiotensin II stimulates the TNF-α signaling pathway and TNF-α upregulates AT1R and its downstream signaling pathway [26,27,28,29]. In addition, TNF-α-mediated apoptosis is associated with the activation of downstream NF-κB signaling pathways, which play vital roles in the control of cell proliferation and death [30].

Consistent with these findings, in our study, the expression of TNF-α mRNA and the AT1R protein was increased in the ureteral obstructed kidney. Furthermore, our in vitro results show that the expression of the AT1R protein and TNF-α mRNA, and the generation of ROS, were increased in TNF-α-treated NRK52E cells. Nuclear translocation of the NF-κB p65 subunit also increased following TNF-α treatment. Therefore, TNF-α treatment resulted in increased cell death and the activation of the apoptotic signaling pathway in rat proximal tubular cells [31].

Overall, our results demonstrate that GPR40 activation rescued cell death and activated the pro-apoptotic signaling pathway in TNF-α-induced injury of NRK52E cells. In addition, the activation of GPR40 inhibited TNF-α-mediated NF-κB activation. The inhibition of GPR40 by a GPR40 antagonist, GW1100, or GPR40 siRNA transfection, promoted the expression of pro-apoptotic proteins by TNF-α treatment. These observations suggest that GPR40 has an anti-apoptotic effect in renal tubular epithelial cells. Our findings raise the possibility that GPR40 might be a novel target for the prevention and treatment of renal cell apoptosis.

## 4. Materials and Methods

### 4.1. Animals

The experimental protocol was approved by the Institutional Animal Care and Use Committee of Chonnam National University Medical School (CNU IACUC-H-2019-11; 8 April 2019). Male 8-week-old C57BL/6J mice were used (Samtako, Osan, Korea). The mice were randomly assigned to the control group and the UUO group, with eight mice each group. UUO was induced by ligation of the left proximal ureter as previously described [19]. The mice had free access to standard chow (Damul Science, Daejeon, Korea) and tap water, and they were sacrificed by decapitation on day 14 after the operation. The kidney was rapidly removed from the animals. The cortex/outer strip of the outer medulla was isolated and stored at −70 °C until used for Western blot analysis and for the reverse transcription polymerase chain reaction (RT-PCR). Western blot analysis and RT-PCR were performed as previously described [5,6,32].

### 4.2. Cell Culture

The NRK52E cells (American Type Culture Collection, Manassas, VA, USA) were treated with TNF-α (20 ng/mL; R&D Systems, Minneapolis, MN, USA) for 24 h in the presence or absence of the GPR40 agonist, GW9508 (10 μM; Cayman Chemical, Ann Arbor, MI, USA), for 1 h prior to the addition of TNF-α, and then harvested for further analysis. The control cells were treated with the vehicle (dimethyl sulfoxide). The NRK52E cells were also treated with TNF-α with or without a 1 h pretreatment with the GPR40 antagonist, GW1100 (10 μM; Cayman Chemical), or GPR40 small interfering (si)RNA (50 nM; ON-TARGET plus Rat Ffar1 (Gene ID: 266607), Item No. L-080051-02-0010, Dharmacon, Lafayette, CO, USA).

### 4.3. Cell Viability Assay

Cell viability was determined using the EZ-CyTox (tetrazolium salt, WST-1) cell viability assay kit (Daeil Lab Service, Seoul, Korea), as previously described [33]. Absorbance at 570 nm was detected using a 96-well microplate reader (BioTek Instruments, Winooski, VT, USA). Cell viability is expressed as the fraction of the surviving cells relative to the vehicle-treated cells.

### 4.4. Preparation of Nuclear and Cytoplasmic Extracts

To prepare nuclear extracts, the cells were lysed using the NE-PER^®^ nuclear and cytoplasmic extraction reagent (Pierce Biotechnology, Rockford, IL, USA) according to the manufacturer’s protocol, as previously described [5]. Briefly, the NRK52E cells were harvested by scraping the cells into a cold phosphate-buffered saline (PBS), pH 7.2, followed by centrifugation at 14,000× *g* for 2 min. After removing the supernatant, 100 µL of ice-cold cytoplasmic extraction reagent I was added to the cell pellets and then incubated on ice for 10 min. The ice-cold cytoplasmic extraction reagent II was then added to the tube and centrifuged at 16,000× *g* for 5 min. The pellet was suspended in 50 µL of ice-cold nuclear extraction reagent, followed by centrifugation at 16,000× *g* for 10 min. Finally, the supernatant containing the nuclear extract was transferred to a new tube and the protein concentration was measured.

### 4.5. ROS Generation

Intracellular ROS generation was measured with a 2′,7′-dichlorodihydrofluorescein diacetate (H_2_DCF-DA) fluoroprobe (Molecular Probes, Eugene, OR, USA). The NRK52E cells were incubated with 5 µM H_2_DCF-DA for 30 min at 37 °C. Then, the cells were washed, collected by centrifugation, and resuspended in PBS. The fluorescence intensity was measured using a FACSCalibur™ flow cytometer (BD Biosciences, San Jones, CA, USA).

### 4.6. Primary Antibodies

Anti-Bax (2772), anti-Bcl-2 (2870), anti-caspase-3 (9662), anti-cleaved caspase-3 (9661), anti-NF-κB p65 subunit (3034), anti-histone H3 (9715), and anti-IκB-α (9242) antibodies were purchased from Cell Signaling Technology (Beverly, MA, USA). The anti-GPR40 and anti-AT1R (SC-1173) antibodies were obtained from Santa Cruz Biotechnology (Dallas, TX, USA). An anti-β-actin (A3854) antibody (Sigma-Aldrich, St. Louis, MO, USA) was used as the control.

### 4.7. Primer Sequences for RT-PCR

Primer sequences for RT-PCR were as follows: for TNF-α, 5′-GTC GTA GCA AAC CAC CAA GC-3′ (forward) and 5′-CTC CTG GTA TGA AAT GGC AAA-3′ (reverse); for GAPDH, 5′-ATC AAA TGG GGT GAT GCT GGT GCT G-3′ (forward) and 5′-CAG GTT TCT CCA GGC GGC ATG TCA G-3′ (reverse).

### 4.8. Immunohistochemistry and Immunofluorescence Staining

The kidneys, fixed in 4% paraformaldehyde, were dehydrated through a graded series of ethanol, embedded in paraffin, sectioned (5 µm), and mounted on glass slides. After deparaffinization and rehydration, antigen retrieval was performed by using Antigen Unmasking Solution (Vector Laboratories, Burlingame, CA, USA). Sections were blocked with 2.5% bovine serum albumin in PBS, incubated with the anti-GPR40 antibody overnight at 4 °C, and then with the appropriate secondary antibody. For immunofluorescence labeling with specific tubular maArkers, the sections were incubated with an Alexa Fluor 568-labeled goat anti-rabbit IgG (1:200 dilution; Invitrogen, Seoul, Korea) secondary antibody after incubation with the anti-GPR40 antibody. Anti-AQP1 and anti-AQP2 antibodies (Alomone Laboratories, Ltd., Jerusalem, Israel) were used as markers for the proximal tubule and for the collecting duct, respectively. The sections were incubated with FITC-conjugated secondary antibodies and the nuclei were counterstained with 4′,6-diamidino-2-phenylindole. Images were captured using an LSM 510 confocal microscope (Carl Zeiss, Jena, Germany).

### 4.9. Statistical Analyses

The results are expressed as means ± standard error of the mean. The statistical significance of the differences was determined by the unpaired t-test or one-way analysis of variance followed by the post-hoc Tukey’s HSD (honestly significant difference) test. The differences were considered statistically significant when the *p* values were *<*0.05, using the GraphPad Prism 6 (GraphPad Software, San Diego, CA, USA).

## 5. Conclusions

The results of our study demonstrate that the decreased expression of GPR40 in the ureteral obstructed kidney is associated with apoptosis via the activation of TNF-α and AT1R. In addition, the activation of GPR40 attenuates TNF-α-induced apoptosis by inhibiting AT1R expression and ROS generation through the regulation of NF-κB signaling pathways.

## Abbreviations

GPR40	G-protein-coupled receptor 40
ROS	reactive oxygen species
NF-κB	nuclear factor-κB
CKD	chronic kidney disease
TNF-α	tumor necrosis factor α
AT1R	angiotensin II type 1 receptor
NRK52E	rat proximal tubular
UUO	unilateral ureteral obstruction
RT-PCR	reverse transcription polymerase chain reaction
AQP	aquaporin
PBS	phosphate-buffered saline
H2DCF-DA	2,7-dichlorodihydrofluorescein diacetate
